# INSIGHT: Evaluation of the Year Four Psychological Medicine Student Placements at HMP Berwyn, North Wales

**DOI:** 10.1192/bjo.2022.408

**Published:** 2022-06-20

**Authors:** Sadia Nafees, Andrea Taylor-Clutton, Simon Newman, Rob Poole

**Affiliations:** 1 Bangor University, Wrexham, United Kingdom; 2 Betsi Cadwaladr University Health Board, Wrexham, United Kingdom

## Abstract

**Aims:**

*Background:* Although social determinants of health (SDOH) are to some extent incorporated within preclinical medical education, little validated educational methodology exists to provide guidance on how to integrate teaching about SDOH within the competency-based training of medical students’ clinical years. This is potentially important. The COVID-19 pandemic has highlighted the importance of SDOH, and social determinants have become topical, with increasing discussion in journals about equity, inequality, and sustainability. Sir Michael Marmot's 2020 review has highlighted these issues. Consequently, the evaluation of medical students’ experience of prison placements is an interesting area to investigate. This will help us to explore their understanding of SDOH, and implications of gained knowledge for their future practice. *Aims:* This study explores changes in year four psychological medicine students’ knowledge and attitude towards SDOH during prison placements.

**Methods:**

A mixed-methods study between Sep 2021 to Apr 2022 recruiting all year four medical students on their psychiatry placement in North Wales. Data collection involves baseline and mid-placement questionnaires and end-of-placement individual interviews to explore their understanding of SDOH.

Thematic analysis will be used to describe students’ reflection on placement satisfaction; explore impacts on trainee doctors and supporting staff; make suggestions to improve placement structure in the future; and evaluate the utility of placements in prison.

**Results:**

Data collection is in progress. However, early indications suggest that students view these placements favourably and find them a helpful learning experience. Preliminary results will be reported at the conference.

**Conclusion:**

We are hopeful that this evaluation will suggest a way forward to raise awareness about SDOH during clinical placements and will give these students confidence in working with socially excluded populations in the future.

**Implication for practise, policy and research:**

Findings of this study may provide exploration of means of capacity building and training with improved knowledge of the SDOH in partnership between the medical school, the local health board and the prison.

We have developed systems and processes to raise awareness of social factors to be considered by medical students in their future practice. These can guide further development of such placements at HMP Berwyn and in other prisons.

This research was funded by Betsi Cadwaladr University Health Board and sponsored by Bangor University in North Wales, UK.

